# Prevalence of Exposure to Environmental Metal Mixtures Among Pregnant Women in the United States National Health and Nutrition Examination Survey (NHANES) 1999–2018

**DOI:** 10.3390/jox15020038

**Published:** 2025-03-01

**Authors:** Patricia Ruiz, Po-Yung Cheng, Siddhi Desai, Mikyong Shin, Jeffery M. Jarrett, Cynthia D. Ward, Youn K. Shim

**Affiliations:** 1 Office of Innovation and Analytics, Agency for Toxic Substances and Disease Registry, Centers for Disease Control and Prevention (CDC), Atlanta, GA 30329, USA; 2 Division of Laboratory Sciences, National Center for Environmental Health, Centers for Disease Control and Prevention (CDC), Atlanta, GA 30329, USA; 3 Oak Ridge Institute for Science and Education, Oak Ridge, TN 37830, USA; 4 Division of Environmental Health Science and Practice, National Center for Environmental Health, Centers for Disease Control and Prevention (CDC), Atlanta, GA 30329, USA

**Keywords:** metals, metal mixtures, cadmium, lead, mercury, pregnancy, trimesters, NHANES

## Abstract

Although exposure to metals remains a public health concern, few studies have examined exposure to combinations of metals. This study characterized prevalent combinations of cadmium (Cd), mercury (Hg), and lead (Pb) in women (n = 10,152; aged 20–44 years) who participated in the U.S. National Health and Nutrition Examination Survey (NHANES) 1999–2018. To explore relative metal exposures within this population, Cd, Hg, and Pb blood levels were dichotomized as “high” and “low” categories using median values to represent the center of the metal concentrations in the study population, not thresholds for adverse health effects. The prevalence of the three metal combinations at “high” levels (singular, binary, tertiary combinations) was calculated. Multinomial logistic regression was used to calculate odds ratios for each combination relative to none of these combinations after adjusting for potential confounders. Among the pregnant women (n = 1297), singular Hg was most prevalent (19.2% [95% CI 15.0–23.3]), followed by singular Cd (14.7% [95% CI 11.2–18.2]), tertiary combination Cd/Hg/Pb (11.0% [95% CI 8.7–13.2]), binary combinations Cd/Pb (9.8% [95% CI 7.4–12.2]), Hg/Pb (9.2% [95% CI 6.5–11.8]), Cd/Hg (7.8% [95% CI 6.0–9.6]), and singular Pb (5.5% [95% CI 4.1–6.9]). We found significantly lower odds of having Cd/Hg/Pb (adjusted odds ratio (adjOR) = 0.49: *p* < 0.001) and Cd/Pb (adjOR = 0.68: *p* < 0.0364) combinations among pregnant women compared to non-pregnant women. The odds of having higher levels of singular Pb were significantly lower (adjOR = 0.31: *p* < 0.0001) in women pregnant in their first and second trimesters (n = 563) than in non-pregnant women (n = 6412), whereas, though nonsignificant, the odds were higher for women pregnant in their third trimester (n = 366) (adjOR = 1.25: *p* = 0.4715). These results indicate the possibility that the fetus might be exposed to higher levels of the metal mixtures due to placental transfer, particularly to Pb, during the early stages of pregnancy. Further research is warranted to understand the relationship between metal combination exposures during pregnancy and maternal and infant health.

## 1. Introduction

Cadmium (Cd), mercury (Hg), and lead (Pb) are among the top 10 on the Agency for Toxic Substances and Disease Registry’s substance priority list, which ranks substances based on their frequency, toxicity, and potential for human exposure at National Priority List sites [1]. Cd, Pb, and Hg persist and bioaccumulate along the food chain and in drinking water, soils, and air, making them a major source of environmental toxicants to humans [2,3,4].

Preterm pregnancy, preeclampsia, low birth weight, and shorter birth length are just a few of the adverse outcomes that Cd, Pb, and Hg have been associated with for pregnant women and infants [5,6,7,8]. Exposures may be more likely to cause harm for pregnant women and fetuses during sensitive developmental time periods in the different stages of pregnancy. In non-pregnant women of reproductive age, exposure to Pb can increase the risk of infertility [9].

While women of reproductive age represent an important target population for the characterization of environmental exposures for women who may become pregnant in the future, body burdens of metal exposures during reproductive age are not generalizable to pregnant women due to physiological, anatomical, and lifestyle changes during pregnancy. Therefore, the identification of metal exposures among pregnant women, together with non-pregnant reproductive-aged women, is of paramount importance to understand the relationships between the environmental exposures and the pregnancy outcomes as well as long-term maternal and child health effects [2,3,4,5].

Pb, Hg, and Cd can cross the placenta and reach the developing fetus during pregnancy [3,4,5,10]. The Cd ion easily moves across biological membranes. Once in the maternal bloodstream, it binds to proteins such as metallothionein and albumin to transport to the placenta [2]. In the placenta, syncytiotrophoblast cells can take Cd and transport it to the fetal side. During pregnancy, Cd interacts with calcium and vitamin D metabolism, increasing Cd absorption and body burden [11]. Blood Cd (BCd) reflects both recent and cumulative exposures [2]. In typical environmental exposure, urinary Cd (UCd) reflects cumulative exposure and Cd concentration in the kidney. There are no specific guidelines for Cd levels during pregnancy. However, high levels of BCd may indicate exposure from sources such as smoking, contaminated food or water, or occupational hazards [2]. UCd levels can be measured to assess long-term exposure. High UCd levels may suggest chronic exposure and potential health risks [2].

Pb exposure during pregnancy primarily occurs through ingestion or inhalation of Pb-contaminated substances such as water, food, or air [3]. Once absorbed into the bloodstream, Pb distribution depends on factors like maternal bone turnover rate and calcium status [12]. Pb stored in bones can be released into circulation during pregnancy due to increase physiological and anatomical demand (e.g., pregnancy-induced bone resorption) [3,12]. Passive diffusion occurs due to Pb’s lipophilic nature, allowing it to pass through cell membranes. Active transport involves binding lead to calcium channels or other transporters on syncytiotrophoblast cells in the placenta. Pb can also bind to proteins like albumin for transport in the maternal bloodstream [3]. Pb metabolism mainly occurs in the liver and is excreted primarily through urine [3]. Blood Pb (BPb) measurement is the preferred method of evaluating Pb exposure and its human health effects because it reflects recent intake and equilibration with stored Pb in other tissues, particularly in the skeleton. Urine Pb (UPb) levels may reflect recently absorbed Pb, though there is greater individual variation in UPb than in BPb [13]. There is no established safe level of Pb exposure during pregnancy, and UPb levels are not commonly used to assess Pb exposure during pregnancy [3].

Hg exposure during pregnancy commonly occurs through consuming contaminated fish or other seafood [4]. The form of Hg determines its toxicokinetics. Once in the maternal bloodstream, elemental Hg and methylmercury (MeHg) bind to proteins like cysteine or glutathione for transport. In the placenta, these forms of Hg are transported via specific transporter proteins [4]. The gastrointestinal tract readily absorbs MeHg (organic form), which crosses the placenta easily [4,14]. In contrast, inorganic Hg in the blood results in limited placental transfer after exposure to elemental Hg (inhalation) and inorganic Hg (ingestion) [15]. Due to its affinity for sulfhydryl groups in proteins, MeHg distributes throughout various tissues, including the brain [4]. It undergoes metabolism mainly in the liver and is eliminated slowly from the body. The total blood Hg concentration in the general population is primarily due to the dietary intake of organic forms, particularly MeHg. Urinary Hg (UHg) consists mainly of inorganic Hg. These distinctions can help interpret blood Hg (BHg) levels in people. Total BHg levels increase with greater fish consumption. UHg levels increase as exposure to elemental and inorganic exposures increase such as from Hg-containing dental fillings, the use of some traditional medicines, the use of Hg-containing skin-lightening creams, or the use of Hg in the workplace [16]. BHg levels above a reference dose value may indicate excessive exposure. UHg levels are not commonly used to assess Hg exposure during pregnancy [4].

The reported associations between Cd, Pb, and Hg body burden and potential health effects underscore the urgent need to comprehend and identify the prevalence of individual and combined metal body burdens in women of reproductive age and during pregnancy. This understanding is crucial for developing effective strategies to mitigate the health risks associated with metal exposure. Several studies conducted among pregnant women revealed socioeconomic disparities in exposure to metals such as Cd, Hg, and Pb [17,18,19]. Previous studies found that Pb exposure in women of childbearing age was associated with demographic, socioeconomic, and environmental factors such as age, race/ethnicity, educational level, poverty, alcohol use, cigarette smoking, serum protoporphyrin level (indicating exposure or poisoning with Pb), and so forth [20,21]. Lower maternal education attainment, father’s occupational status, and parental smoking at home were also associated with increased blood Pb levels among children [22].

Epidemiological data are accumulating on the adverse health effects of metal exposures on the health of the fetus and the mother. However, few studies have investigated the concentrations of metal combinations in urine or blood during pregnancy, particularly in each trimester. Furthermore, the health risks from exposures to the combination of metals Cd, Hg, and Pb during the trimesters of pregnancy or at any time during pregnancy are still unknown [23,24].

This study aimed to identify prevalent co-exposures to Cd, Hg, and Pb in blood and urine among women of reproductive age who participated in the U.S. National Health and Nutrition Examination Survey (NHANES) 1999–2000 to 2017–2018 and to characterize the co-exposures during early pregnancy (first and second trimesters) and late pregnancy (third trimester) by comparing them with those in non-pregnant women of reproductive age.

## 2. Materials and Methods

### 2.1. Data

CDC’s National Center for Health Statistics (NCHS) conducts the NHANES, a complex cross-sectional survey designed to produce a sample representative of the U.S. population based on age, sex, and race/ethnicity. The NHANES combines questionnaires, medical examinations, and laboratory biomonitoring methods to determine the health and nutritional status of the civilian, noninstitutionalized general U.S. population. Data from about 5000 participants are collected annually through interviews, surveys, physical examinations, and clinical specimens (samples of human tissues or fluids). Data are released in 2-year cycles. NHANES assigned each participant a sample weight to account for his or her probability of selection as well as for non-response. Subsample weights, modified for combined cycles, and design variables (including survey weights, strata, and primary sampling units) were used to account for the NHANES’s complex sample design. Taylor series linearization methods were used for variance estimation [25]. The NHANES protocol is in compliance with the HHS Policy for Protection of Human Research Subjects (45 CFR part 46). It was approved by the NCHS Research Ethics Review Board.

### 2.2. Study Population

Women of childbearing age (20–44 years) from the NHANES 1999–2018 were included in this study. Pregnancy information was collected from (1) demographic data (variable RIDEXPRG: pregnancy status at the time of examination at mobile examination center), (2) pregnancy urine test data (variable URXPREG: pregnancy test result), and (3) reproductive health data (variable RHD143: “Are you pregnant now?” and variable RHD152: “what month of pregnancy are you in?”). The pregnant woman was determined by RIDEXPRG = 1 (yes), URXPREG = 1 (positive), or RHD143 = 1 (yes). Women with the trimester status (n = 1009) were categorized by using RHD152 (first trimester: first through third months; second trimester: fourth through sixth months; third trimester: seventh through ninth months).

The NHANES data only provide variables (RIDEXPRG, URXPREG, and RHD143) to define pregnant women, who were limited to ages 20–44 year-old due to disclosure risk. NHANES population does not include individuals under the age of 20 year-old in the reproductive age group. However, it covers a significant portion of women compared to national demographics. According to the national demographics (NCHS birth data in 2022), the age group from 20 to 44 years old represented 95.7% of births to childbearing age in 2022 in the United States [26].

The 20–44-year-old age range is widely acknowledged to include the key reproductive years for women, with fertility rates generally higher than any other reproductive age. This age range is associated with higher fertility rates, regular menstrual cycles, lower pregnancy complications, and higher success rates [26].

To increase the sample size of the pregnant women, we combined the NHANES cycles from 1999–2000 to 2017–2018 for the analysis of blood metal levels (Cd, Hg, and Pb) of pregnant women (N = 1297) vs. non-pregnant women (N = 8855). The data on trimester status of the pregnant women available from the cycles 1999–2000 to 2011–2012 were used for the analysis of blood levels of the metals in pregnant women with trimester status (first and second: n = 615, 3rd: n = 394) vs. non-pregnant women (N = 6966). Urinary levels of the metals were measured from a one-third sample and available for the cycles from 2003–2004 to 2017–2018, which were used for the analysis of the metal exposure in pregnant women (N = 285) vs. non-pregnant women (N = 2756).

NHANES started collecting blood ethyl and methyl Hg data in 2011. No blood Hg speciation data were available before 2011. For blood inorganic Hg data in the NHANES, the detection rates were below 50% for most cycles. Due to these reasons, we did not focus on blood Hg speciation in this analysis.

### 2.3. Analytical Measures

Hg was measured in urine during the NHANES 1999–2004 using flow injection cold vapor atomic absorption spectroscopy (FI-CVAA) [27]. Inductively coupled plasma dynamic reaction cell mass spectrometry (ICP-DRC-MS) was used to measure Hg in urine during the NHANES 2005–2012 [28] and the NHANES 2013–2018 [29]. Hg was measured in blood during the NHANES 1999–2002 using FI-CVAA [30]. Pb and Cd were measured in blood during NHANES 1999–2002 using graphite furnace atomic absorption spectroscopy (GFAAS) [31]. ICP-MS was used for testing Pb, Cd, and Hg in blood during the NHANES 2003–2010 [32] and 2011–2018 [33,34]. Pb was measured in urine during the NHANES 1999–2018 using ICP-MS [35,36]. ICP-MS was used to test Cd in urine during the NHANES 1999–2002 [37,38], and ICP-DRC-MS was used during the NHANES 2003–2018 [35,39]. Serum cotinine is measured by using an isotope-dilution high-performance liquid chromatography/atmospheric pressure chemical ionization tandem mass spectrometric (ID HPLC-APCI MS/MS) method [40]. Urinary creatinine is measured by using an enzymatic method [41]. Creatinine-adjusted metal concentrations in urine account for variations in urine dilution. The creatinine level was used to adjust the urine samples (value of urine sample/creatinine level × 100). Since urine volume can fluctuate, creatinine correction helps standardize the measurement of metal levels. Thus, creatinine-adjusted metal levels provide a more reliable assessment of exposure, especially when comparing samples from different individuals or monitoring changes over time. Those adjusted values were used to prepare the urinary metal prevalence table (Supplementary Table S3). Because the sample size of the pregnant women in the urinary data was small, no further analysis was performed for the urinary data.

Limit of detection (LOD) in analytical methods represents the lowest concentration of a chemical of interest in a sample that can be reliably distinguished from a blank (where no chemical of interest is present) and the threshold down to which quantification was performed. In statistical analyses, chemical concentrations below the LOD are frequently imputed or replaced with a fixed value, like half the LOD, the LOD divided by the square root of 2, or zero. In our analysis, the imputed values were calculated as LOD divided by the square root of 2. The LOD values for blood Cd, Hg, and Pb in our analysis are presented in Supplementary Table S4a,b.

### 2.4. Statistical Methods

#### 2.4.1. Geometric Means and Percentiles

We calculated category-specific geometric means and percentiles using SUDAAN version 11.0.3 (Research Triangle Institute, Research Triangle Park, NC, USA). SUDAAN uses sample weights and calculates variance estimates that account for the complex survey design. We estimated the 95% confidence intervals for geometric means based on the Taylor series linearization method (SUDAAN user manual, 2002) [42]. We adapted confidence intervals for percentiles from the methods of Korn and Graubard (1998) and Woodruff (1952) [43,44].

#### 2.4.2. Prevalence

The dichotomization of continuous variables is a common practice in research and data analysis [45]. Potential joint toxic effects from multiple metals coexisting in the human body and their effective dose levels are not clearly understood. Such studies are resource-intensive and time-consuming because the available data are sparse. For future research it is, therefore, necessary to prioritize metal combinations based on their prevalence in human populations [46]. In the absence of clear understanding of dose–response relationships for the co-existing metals, we opted to use the median values as cut points to identify common combinations in the population. Higher cut points, such as the 95th percentile, will not represent the common exposure levels of the general population.

Our prevalence analyses included two parts. For blood or urine levels of Cd, Hg, and Pb, we performed the following procedures to calculate metal prevalence for each group (non-pregnant or pregnant). A dichotomous variable as “high” and “low” was created separately for each of the three metals, indicating whether a person’s metal concentrations were greater than or equal to the respective medians of the entire study population for the time frame of the study. We used the median values to represent the center of the metal concentrations in the study population, not adverse health effect threshold levels. The results of biomonitoring studies need be compared with health references values in order to provide useful information in the application of risk management strategies.

All possible unique scenarios of the three metals (i.e., None, Cd, Hg, Pb, Cd/Hg, Cd/Pb, Hg/Pb, and Cd/Hg/Pb) were identified, where each metal concentration was at or above the population median. The prevalence of unique combinations (singular, binary, tertiary) was calculated for the three metals. For the second part of the analysis, we studied metal prevalence in blood samples for women by trimester of pregnancy. For trimester status, the data were compared for three groups (non-pregnant (N = 6966), first and second trimester (N = 615), and third trimester (N = 394)). For each group, we performed the same procedures to calculate the metal prevalence, as noted in the first part of the analysis.

#### 2.4.3. Factors Associated with Each Combination

Multivariate analysis was performed with multinomial logistic regression to identify the independent variables associated with each mixture combination (specific unique combinations were compared to “None”). Those independent variables were chosen with a priori knowledge. They included pregnancy status, age (20–29 years, 30–44 years), race/ethnicity (Mexican American, non-Hispanic Blacks, non-Hispanic Whites, other races), income to poverty ratio (≤1.85, 1.85–3.5, >3.5) [47], and serum cotinine level (<0.015 ng/mL, 0.015–10 ng/mL, ≥10 ng/mL). For the time frame of the NHANES 1999–2000 to 2017–2018 data, the pregnancy status is categorized as non-pregnant or pregnant. For the time frame of the NHANES 1999–2000 to 2011–2012 data, the pregnancy status is classified as not pregnant, first and second trimesters, or third trimester. The logistic analysis did not include the urinary metal data because of the small sample size.

We combined the first and second trimesters to increase the sample size. Pregnant women experience physiological changes in the first and second trimesters more than in the third trimester. These changes include increased blood volume, changes in cardiac output, fluctuating hormone levels, altered immunological response, and changes in metabolism.

Major organ systems develop in the fetus during the first trimester, but certain organ systems continue to develop and mature during the second trimester. In addition, the placenta starts to develop during the first trimester and progressively takes over the mother’s and fetus’s ability to exchange nutrients and produce hormones. The placenta is completely developed during the second trimester and is essential to the fetus’s growth and development. Therefore, given the small sample sizes of pregnant women in the first and second trimesters, we opted to combine the first and second trimesters groups.

## 3. Results

### 3.1. Characteristics of the Study Population

A total of 10,152 women aged 20–44 years were selected from the NHANES 1999–2018 data.

For the NHANES 1999–2018 data, the average age was 32.4 years for non-pregnant women and 28.4 years for pregnant women (*p* < 0.0001) (Supplementary Table S1). Race/ethnicity, poverty income ratio, and serum cotinine levels were significantly different between pregnant and non-pregnant groups. The geometric means and median of the concentrations of blood Cd, Hg, and Pb for non-pregnant and pregnant women are shown in Supplementary Table S2.

### 3.2. Metals Prevalence

In 17.4% [95% CI 16.1–18.7] of the U.S. non-pregnant women, none of the three metals were detected in blood at or above the respective population medians (Table 1). Of the U.S. non-pregnant women, 29.9% had only a single metal detected (Cd: 10.0% [95% CI 9.2–10.8], Hg: 13.4% [95% CI 12.3–14.5], and Pb: 6.5% [95% CI 5.8–7.2]); 32.6% had two metals (Cd/Hg: 7.6% [95% CI 6.8–8.4], Cd/Pb: 15.6% [95% CI 14.4–16.8], and Hg/Pb: 9.4% [95% CI 8.5–10.3]); and 20.0% [95% CI 18.8–21.3] had all three metals (Cd/Hg/Pb). In contrast, in 22.9% [95% CI 19.0–26.8] of U.S. pregnant women, none of the three metals were detected in blood at or above the respective population medians. In 39.4% of the U.S. pregnant women only a single metal (Cd: 14.7% [95% CI 11.2–18.2], Hg: 19.2% [95% CI 15.0–23.3], and Pb: 5.5% [95% CI 4.1–6.9]) was detected; 26.8% of the U.S. pregnant women had two metals (Cd/Hg: 7.8% [95% CI 6.0–9.6], Cd/Pb: 9.8% [95% CI 7.4–12.2], and Hg/Pb: 9.2% [95% CI 6.5–11.8]); and 11.0% [95% CI 8.7–13.2] of the U.S. pregnant women had three metals (Cd/Hg/Pb). Among the pregnant women (n = 1297), singular Hg was most prevalent (19.2% [95% CI 15.0–23.3]), followed by singular Cd (14.7% [95% CI 11.2–18.2]), tertiary combination Cd/Hg/Pb (11.0% [95% CI 8.7–13.2]), binary combinations Cd/Pb (9.8% [95% CI 7.4–12.2]), Hg/Pb (9.2% [95% CI 6.5–11.8]), Cd/Hg (7.8% [95% CI 6.0–9.6]), and singular Pb (5.5% [95% CI 4.1–6.9]). The overall prevalence estimates of Pb alone or in combination would be 35.5% in pregnancy compared to 51.5% in non-pregnant; Cd would be 43.3% vs. 53.2%, and Hg would be 47.2% vs. 50.4%.

None of the three metals were detected in urine at or above the respective population medians in 20.8% [95% CI 18.6–23.0] of the U.S. non-pregnant women (Supplementary Table S3). Also, the results show that pregnant and non-pregnant have the same distributions, and pregnant women do not excrete more metal in urine than non-pregnant women (Supplementary Figure S1).

### 3.3. Between Trimesters Prevalence

Table 2 shows the prevalence of metals in pregnant women at various trimesters in the U.S. population. For the women in the first and second trimesters (combined), 26.4% [95% CI 19.3–33.4] had none of the three metals detected in blood at or above the respective population medians, 38.2% had only a single metal (Cd: 14.9% [95% CI 10.7–19.1], Hg: 19.9% [95% CI 13.5–26.2], and Pb: 3.4% [95% CI 2.1–4.8]), 26.4% had two metals (Cd/Hg: 9.7% [95% CI 5.7–13.7], Cd/Pb: 11.4% [95% CI 7.1–15.7], and Hg/Pb: 5.3% [95% CI 1.4–9.2]), and 9.0% [95% CI 5.1–12.9] had all three metals.

For the women in the third trimester, 21.7% [95% CI 15.0–28.3] had none of the three metals detected in blood at or above the respective population medians; 46.5% had only a single metal (Cd: 16.5% [95% CI 9.6–23.3], Hg: 20.1% [95% CI 12.8–27.5], and Pb: 9.9% [95% CI 5.9–14.0]), 19.9% had two metals (Cd/Hg: 6.9% [95% CI 3.2–10.5], Cd/Pb: 6.8% [95% CI 4.2–9.4], and Hg/Pb: 6.2% [95% CI 3.6–8.8]), and 11.9% [95% CI 6.0–17.9] had all three metals.

### 3.4. Factors Associated with Exposure to Cd, Hg, Pb, and Their Combinations

Table 3 presents multinomial logistic regression results for U.S. women aged 20–44 years old who participated in the NHANES 1999–2018. For Pb, Cd/Pb, and Cd/Hg/Pb, pregnant women had significantly lower odds for having a blood metal level at the median or higher relative to non-pregnant women, after adjusting for covariates determined a priori (age, race/ethnicity, income to poverty ratio and serum cotinine). Pregnant women had higher odds of having a median or higher blood Cd level (adjOR = 1.48 [95% CI 1.02–2.15]), after adjusting for other factors. Women aged 30–44 years had significantly higher odds of having a blood metal level at the median or higher levels, especially for Cd/Hg/Pb (adjOR = 3.04 [95% CI 2.40–3.84), than women aged 20–29 years, after adjusting for their pregnancy status and other covariates.

Relative to non-Hispanic Whites, Mexican Americans and other races had a significantly higher odds for having metals (Cd, Cd/Hg, Cd/Pb, Hg/Pb, Cd/Hg/Pb) in blood at the median or higher level. For Hg, Cd/Hg, Hg/Pb, and Cd/Hg/Pb, non-Hispanic Blacks had a significantly higher odds for having a median or higher blood metal level than non-Hispanic Whites.

Relative to women with a low poverty income ratio (PIR ≤ 1.85), women with middle poverty income ratios (PIR: 1.85–3.5) had 0.65, 95% CI [0.48–0.86], times lower odds for having a blood Cd or Cd/Pb level at the median or higher. Relative to women with PIR ≤ 1.85, women with a PIR of >3.5 had more than two times higher odds for having a blood Hg (adjOR = 2.07 [95% CI 1.59–2.68]), Cd/Hg (adjOR = 2.52 [95% CI1.82–3.51], Hg/Pb (adjOR = 2.47 [95% CI 1.89–3.22]), and Cd/Hg/Pb (adjOR = 2.24 [ 95% CI 1.75–2.86]) level at the median or higher level.

**Figure 1:**
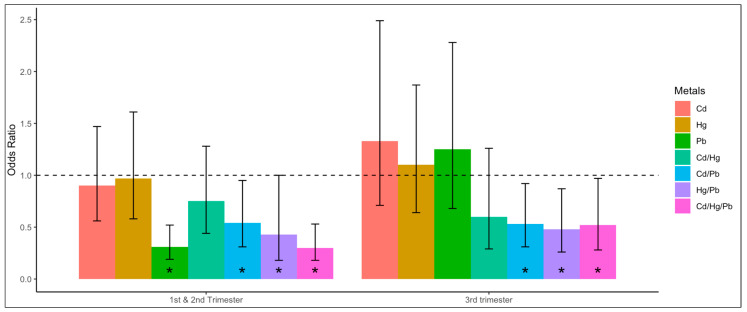
Associations between pregnancy status and individual unique combinations of blood cadmium (Cd), mercury (Hg), and lead (Pb) among U.S. women ^ aged 20–44, National Health and Nutrition Examination Survey (NHANES), 1999–2012. Odds ratios were calculated using multinomial logistic regression analysis. The dependent variable was a unique combination of the metals (none, Cd, Hg, Pb, Cd/Hg, Cd/Pb, Hg/Pb, Cd/Hg/Pb) detected at or above the respective median levels. The independent variable was the pregnancy status (non-pregnant, first and second trimesters, third trimester; non-pregnant as reference group). Odds ratios were adjusted for age group, race/ethnicity, poverty income ratio, and serum cotinine level. ^ Sample size n = 7341 (6412 non-pregnant women, 179 pregnant women in the first trimester, 384 pregnant women in the second trimester, 366 pregnant women in the third trimester). * *p* < 0.05.

**Figure 2:**
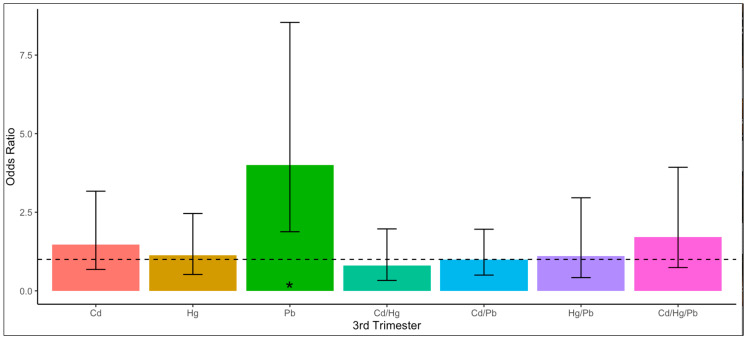
Associations between trimesters and individual unique combinations of blood cadmium (Cd), mercury (Hg), and lead (Pb) among U.S. pregnant women ^, National Health and Nutrition Examination Survey (NHANES), 1999–2012. Odds ratios were calculated using multinomial logistic regression analysis. The dependent variable was the unique combination of the metals (none, Cd, Hg, Pb, Cd/Hg, Cd/Pb, Hg, Pb, Cd/Hg/Pb) detected at or above the respective median levels. The independent variable was trimester categorized by first and second trimester versus third trimester. The reference group was the first and second trimesters. Odds ratios were adjusted for age group, race/ethnicity, poverty income ratio, and serum cotinine level. ^ Sample size = 929 (563 pregnant women in the first trimester and second trimester, 366 pregnant women in the third trimester). * *p* < 0.05.

Relative to women with serum cotinine levels <0.015 ng/mL, women with serum cotinine levels of 0.015–10 ng/mL had 1.35 [95% CI 1.01–1.80] times higher odds, and those with ≥10 ng/mL levels had 34.3 [95% CI 21.1–55.9] times higher odds for having a blood Cd level at the median or higher level. Relative to women with serum cotinine levels <0.015 ng/mL, women with serum cotinine levels of 0.015–10 ng/mL had a 1.59 [95% CI 1.15–2.20] times higher odds, and those with levels ≥10 ng/mL had a 2.76 [95% CI 1.33–5.69] times higher odds for having a blood Pb level at the median or higher level. Relative to women with low serum cotinine levels, women with higher serum cotinine levels had higher odds of having a blood metals level at the median or higher level, except for Hg. The odds of having singular Cd in blood levels at or above the median were significantly higher in pregnant women than in non-pregnant women (Table 3). The odds of having singular Pb blood levels at or above the median were significantly lower among women in the first and second trimesters compared to non-pregnant women (Figure 1). The odds of having singular Pb blood levels at or above the median among pregnant women were significantly higher in the third trimester than in the first and second trimesters (Figure 2).

## 4. Discussion

This analysis used NHANES data (1999–2018) to characterize exposure to combinations of Cd, Hg, and Pb among women aged 20–44 in the United States. To our knowledge, this is the first analysis to estimate exposure to Cd, Hg, and Pb and their combinations during pregnancy and between trimesters. The average blood levels for all three metals have declined substantially [48]. The awareness of toxic effect of metal exposures (e.g., smoking cessation campaigns) may have led to the declines of the blood levels of these metals. We found high detection rates for all three metals measured in blood, ranging from 70.4 to 96.0% in pregnant women and 81.1–99.3% in non-pregnant women; blood Cd had the lowest detection rates in both groups, and blood Pb had the highest. In looking at all possible combinations of the three metals detected above their corresponding median value among pregnant women, the most prevalent combination was singular Hg, followed by singular Cd, tertiary combination of Cd/Hg/Pb, and binary combination of Cd/Pb. Both Cd and Pb drove the difference between the blood concentrations of Cd/Hg/Pb in older vs. younger women (odds ratios for older women having a higher metal level compared to younger women were 1.30 and 1.44 and were statistically significant for Cd and Pb, respectively).

### 4.1. Cd Exposure During Pregnancy

In this analysis, after adjusting for other covariates (age, race/ethnicity, PIR, and serum cotinine levels), Cd was the only metal or metal combination that had significantly higher odds for being present at blood concentrations at or above the median in pregnant women than in non-pregnant women.

Our results are consistent with a previous study by Ruiz et al. that reported that compared to men, women of reproductive age often have higher amounts of Cd in their blood and urine [49]. Pregnant women’s intake of prenatal micronutrients compared to non-pregnant women can also play a role; it is known that, particularly, iron, manganese, and zinc, appear to have an impact on intestinal Cd uptake either directly or indirectly [2]. During pregnancy, Cd interacts with calcium and vitamin D metabolism, increasing Cd absorption and body burden [11]. Due to Cd’s chemical similarity with zinc and calcium, it can disrupt the physiological functions of both. The placenta is a partial barrier for Cd compared to other metals such as Pb and Hg [2]. Cd has been reported to accumulate in the placenta, which limits the transplacental transfer to the fetus [21]. It is very likely that metallothionein is responsible for the placental storage of Cd.

During pregnancy, Cd exposure usually results from inhaling cigarette smoke or consuming contaminated food or water [2]. Fiber-rich foods and medicinal plants might be a noteworthy source of Cd exposure [50,51]. Shimbo et al. found rice to be a significantly greater source of Cd exposure than wheat flour [52], suggesting greater dietary exposure in countries or populations reliant on rice as a staple. In this context, additional research is required to identify the sources of Cd exposure in pregnant and non-pregnant women in the U.S., the role of increased uptake of essential nutrients (e.g., Zn, Fe, Ca) during pregnancy, and the impact of Cd exposure on maternal and infant health.

A study conducted in China found a significant inverse correlation between first-trimester maternal Cd levels and birth weight in girls [53]. Their results suggest that higher Cd exposures during early pregnancy could be associated with low birth weight and a higher risk for its accompanying complications. Additionally, Sabra et al. reported that maternal serum levels of Cd were higher in the small for gestational age than in those appropriate for gestational age [8]. Higher levels of Cd exposure during pregnancy have also been associated with preterm birth [54].

### 4.2. Pb Exposure During Pregnancy

This study found the odds of having maternal blood Pb concentrations at or above the median to be lower in pregnant women than in non-pregnant women. These differences could be due to the physiological and anatomical changes during pregnancy, demographic differences in who becomes pregnant and who does not, as well as parity number [3]. During pregnancy, maternal plasma volume increases, which could contribute to lower concentrations of Pb in early pregnancy [55]. Calcium absorption also increases significantly to meet the needs of both the mother and the developing fetus [3,56]. This increase is primarily due to enhanced intestinal absorption, which is mediated by higher levels of Vitamin D. As the pregnancy progresses and the body is put under increasing physiological demands, calcium is mobilized from the bones, and, subsequently, Pb storage in bones is co-mobilized with calcium, particularly during late pregnancy [57]. Maternal calcium supplement intake might help reduce circulating blood Pb levels, thereby reducing Pb levels in pregnant women compared with non-pregnant women [58].

Pb exposure during late pregnancy might be particularly important because the third trimester is a period of increased fetal growth and need for nutrients such as calcium and vitamin D [24,57]. Pregnant women with low calcium intake may absorb more Pb as the body increases calcium absorption to meet the increased demand, inadvertently increasing Pb absorption [57]. Ensuring sufficient calcium intake can help mitigate this risk by reducing the body’s need to mobilize calcium from the mother’s bones, which may release stored Pb from the mother’s bones [57]. Our study showed the odds of having blood Pb at levels at or above the median were higher during the third trimester than in the first and second trimesters. According to Barltrop’s studies, fetal–maternal placental transfer of Pb begins in the 13th week of pregnancy and continues until childbirth, with Pb content increasing proportionally as the fetus’s organs grow [59]. It is most likely a simple diffusion, which is the main method of Pb transmission across the placenta. Maternal and neonate umbilical cord blood Pb levels are highly correlated [60], even at low concentrations [11], emphasizing the need to address sources of fetal Pb exposure. Pb levels in the umbilical cord blood directly affect the concentration of Pb in the developing fetus tissues, particularly the brain tissue. When the fetus’s organs grow, the Pb content also grows accordingly. As a result of the immaturity of the blood–brain barrier, which makes it easier for Pb to enter the brain, the developing fetus’s brain structures can still be directly damaged. Nutritional deficiencies of calcium worsen the degenerative effects of Pb and increase Pb absorption [61].

Work by Renzetti et al. on the PROGRESS cohort (pre-birth cohort based in Mexico City) revealed significant negative associations between third-trimester maternal blood Pb and the child’s height and weight at age 4–6 years [62]. Higher Pb exposures during this time might adversely affect growth outcomes. Earlier exposure might also pose a risk. A study conducted in China discovered Pb exposure during early pregnancy to be associated with lower birthweight, possibly via disruption of the balance of maternal thyroid hormones [63]. Furthermore, early pregnancy exposures to Pb have been associated with lower intelligence in children [64]. Prenatal Pb exposure has also been associated with lower motor functioning in infants and children [65], as found in a study conducted on mothers across the Navajo Nation who had community exposure to a variety of metals from abandoned uranium mines [66]. Native American mothers are also likely to have increased Pb absorption due to calcium and vitamin D deficiencies [56], making their children vulnerable to prenatal exposure.

Examinations of the Canada-based Maternal-Infant Research on Environmental Chemicals (MIREC) cohort have further revealed positive associations between background-level maternal blood Pb and other adverse pregnancy outcomes. Fisher et al., for instance, found that maternal blood Pb levels, like those in our study, were associated with preterm birth and spontaneous preterm birth [67]. On the other hand, analysis of the MIREC cohort by Borghese et al. showed an association between third-trimester maternal blood Pb and preeclampsia; blood Pb levels were similar to those we reported [6].

Shih et al. used data about mother–infant pairs from the National Children’s Study and found mostly sex-specific associations between certain heavy metals and birth outcomes [7]. Maternal blood Pb levels (overall median: 0.34 ug/dL) appeared to be lower than those in our study and were found to be inversely associated with birthweight, birth length, head circumference, and gestational age in female infants. Shih et al. also found maternal blood Hg levels (overall median for total Hg: 0.58 ug/L) that were lower than our study and were inversely associated with birth weight and ponderal index; birthweight had a particularly stronger association among female infants.

Thomas et al. examined the MIREC cohort whose median blood levels of Cd, Hg, and Pb were comparable to or lower than those in our study. They found a slight increase in the risk of babies being small for gestational age when comparing extreme tertiles of blood Hg. Cd and Pb did not show any correlation [68].

### 4.3. Combined Exposure to Cd/Pb or Hg/Pb During Pregnancy

As scientific knowledge expands, it becomes increasingly evident that more than studying the effects of single chemicals on human health is needed to fully understand the complexities of real-life exposure scenarios. Future research can prioritize investigating the combined effects of multiple chemical exposures to address this limitation. One key direction is to develop advanced analytical techniques capable of simultaneously detecting and quantifying a wide range of chemical mixtures. This will enable scientists to assess chemical combinations’ interactions and synergistic effects in a realistic exposure pathway.

In our study, the odds for having concentrations of Cd/Pb or Cd/Hg/Pb metal combinations at or above the median were lower in pregnant women in any trimester than in non-pregnant women after adjusting for age, race/ethnicity, PIR, and serum cotinine levels.

We also found the odds of having maternal blood concentrations of Hg/Pb at or above the median to be lower in women in both the first and second trimesters and third trimesters compared to non-pregnant women. These findings support the reported mechanism of the individual metals being deposited in women’s placentas and transferred from mother to fetus.

Co-exposure to Hg/Pb during late pregnancy can negatively affect the neurodevelopment of the infant, as found in a study examining neurodevelopment at 6 months using mental development index scores [69]. Shah-Kulkarni and colleagues also found potential evidence of interaction between Pb/Hg levels in late pregnancy affecting mental and psychomotor developmental indices. They found that exposure to Hg during late pregnancy appears to exacerbate the negative effect of Pb on neurodevelopment at 6 months. An examination of the offspring of northern Tanzanian women living in areas with artisanal gold mining activities found that co-exposures to high concentrations of Hg/Pb increased adverse effects on the infants’ neurodevelopment [70]. Prenatal and postnatal exposure to these heavy metals has further been found to be associated with autism [71].

Due to their ability to cross the placenta, the absorption, distribution, metabolism, and excretion of Pb, Cd, and Hg can have significant implications for the mother and the developing fetus, making this a crucial study area. During pregnancy, the absorption of Pb, Cd, and Hg may increase due to changes in gastrointestinal motility and increased blood flow to the gut [72,73]. Furthermore, physiological and anatomical changes during pregnancy can alter the distribution of Pb, Cd, and Hg in the body. Pregnancy-related hormonal changes can influence the metabolism of these metals. Hormone levels increase during pregnancy, affecting how these metals are metabolized [74,75]. The mother’s body adaptations during pregnancy may have reduced her blood levels of these metals through the placental fetal–maternal transport mechanisms. This transfer helps decrease exposure for the mother but increases the potential risk for fetal exposure. The lower metal blood levels observed in pregnant women than in non-pregnant women in our study provide a hypothesis for future exploration of Pb, Cd, and Hg transplacental transport mechanisms.

Assessing the health effects of multiple metal exposures during pregnancy and differences between trimesters poses a lot of complexity involved in evaluating interactions between various metals and their combined effects on human health. The interaction between the metal combinations can result in additive, synergistic, or antagonistic effects, making it difficult to accurately predict the overall impact on maternal and infant outcomes. Furthermore, standardized methodologies for assessing chemical mixture exposures are lacking. Each metal has its unique toxicological profile and exposure pathway, making it challenging to establish a comprehensive framework for evaluating their combined effects during pregnancy and postnatal exposure. To accurately assess the potential risks posed by metal combination exposure during pregnancy, further research is needed.

### 4.4. Heavy Metal Mixed Exposure (Cd, Hg, and Pb) and Sociodemographic Factors

In addition to pregnancy status, we found sociodemographic and lifestyle factors to be associated with higher levels of metals and metal combinations among U.S. women of childbearing age. For instance, women aged 30–44 years had higher odds of having all metals and metal combinations (except Hg) at concentrations at or above the median as compared to women aged 20–29 years. That finding is confirmed by previous NHANES studies on women of child-bearing age and on the general U.S. population: Bulka et al. found that older age was positively associated with higher toxic metal concentrations in women of childbearing age, and Shim et al. reported significantly higher odds of heavy metal combinations in men and women that were ten years older than those that were younger [76,77]. Furthermore, we found women with a PIR > 3.5 to be at higher odds for having Hg and Hg-containing combinations at concentrations at or above the median than were women with a PIR ≤ 1.85 (adjusted ORs, 2.07–2.52). Mahaffey et al. examined blood Hg distributions in women of childbearing age in the United States (NHANES 1999–2004) and similarly found that women with higher incomes had higher blood Hg levels associated with eating more fish [78]. In addition to eating more seafood, women with a higher income might have easier access to dental amalgams or skin-lightening products, both of which have been linked to Hg exposure [79,80]. In keeping with previous research citing cigarette smoke as the major source of Cd and Pb exposure [81], serum cotinine levels ≥10 ng/mL were associated with significantly increased odds for having Pb or Cd-containing metal combinations at or above the median.

Our results suggest that race/ethnicity is another factor associated with mixed metal exposure. Mexican Americans, non-Hispanic Blacks, and other races had significantly higher odds of having most of the metals and metal combinations at blood levels at or above the population median than non-Hispanic White women. Bulka et al. used the NHANES data to study toxic metal exposures among U.S. women of reproductive age. They found that Mexican Americans, non-Hispanic Blacks, and women of other races had higher blood and urine concentrations of toxic metals such as Cd, Hg, Pb, and arsenic (As) [77]. These race/ethnic groups may be more exposed to certain metals because they reside in more polluted areas or near industrial locations. These groups are more likely to have occupational and socioeconomic variables that increase their exposure to toxic metals [82,83]. Additionally, a higher proportion may experience limited access to resources, healthcare, and education, which may result in higher exposure and lower ability to mitigate the effects of these metals. Furthermore, dietary differences may be caused by the eating of specific foods that may have higher concentrations of these metals [84]. A study conducted in the Northeastern United States to examine racial/ethnic and neighborhood disparities in metal exposures during pregnancy found Black, Black Hispanic, and White Hispanic women to have significantly higher urinary metal concentrations than White women in the cohort [82]. Another study found that areas in the Southeastern United States that had a greater proportion of persons from racial and ethnic minority populations had increased exposure to heavy metals such as Cd, As, and Pb among those populations [83].

These results suggest that increasing awareness of harmful exposures and focusing on groups that experience higher levels of environmental risk could help reduce in utero exposures to toxic metal mixtures for the fetus and in women of reproductive age as they are vulnerable to various environmental, nutritional, and lifestyle factors that can impact their health during this life stage.

### 4.5. Strengths and Limitations

Our results offer a starting point for deciding how best to prioritize efforts to evaluate potential negative health consequences and inform body burden levels for further studies of the metal combinations during pregnancy. Combining multiple NHANES cycles allowed for a larger sample size and thereby increased statistical power. To our knowledge, this is the first study to stratify the prevalence of Cd, Hg, and Pb and their combinations by trimester of pregnancy.

When someone is exposed to metals, either sequentially or simultaneously, metal toxicity can occur through many different biological pathways. The nature and degree of toxicity may vary based on the metals exposed together, different exposure pathways (e.g., route and duration), and the metals’ unique toxicokinetic and toxicodynamic properties. Also, each population and individual differ in their susceptibility to toxicity. Additional factors, such as diet, geographical location, and occupation, are available in the NHANES data but were not included in this analysis; these factors can be included in future studies.

The cross-sectional nature of the study lends itself to several limitations, including recall bias and misinterpretation, which can lead to misclassification of trimester status. The cross-sectional design may also limit the ability to control for confounding adequately. Thus, differences in metal prevalence between the different groups (e.g., pregnant vs. non-pregnant, third trimester vs. non-pregnant) may have been due to residual confounding. Furthermore, physiological and anatomical differences between the various periods of time during pregnancy, within the stages of gestation, and non-pregnant time periods are another aspect that must be considered. Additionally, the discrete nature of trimester categorization and the dichotomization of the blood metal levels could also result in a loss of statistical power due to reduced data granularity. Furthermore, our analysis treats each of the three metals as equipotent, but they tend to have differing toxicities. Factors such as synergistic or antagonistic relationships between the metals, dose–response relationships, and limitations in the exposure quantification must also be considered to further elucidate the implications of our results. More studies are urgently required to examine how environmental, dietary, lifestyle, and/or cultural behaviors affect maternal and fetal Cd, Hg, and Pb exposures at and above background levels. Once these risks have been identified, preventive measures should be implemented to reduce or eliminate the exposure of a fetus to these metals or their combinations during pregnancy. Our study used the median values to represent the center of the metal concentrations in the study population, not adverse health effect threshold levels.

The NHANES oversampled pregnant women for the 1999–2006 survey periods, and more pregnant women were ascertained during these periods. The findings may tend to represent the results from these survey periods.

## 5. Conclusions

Among non-pregnant and pregnant women, prevalent combinations of Cd, Hg, and Pb above population medians were identified using the NHANES data. The odds of having Cd/Pb, Hg/Pb, or Cd/Hg/Pb metal combinations at or above the median blood levels were significantly lower in pregnant women in any trimester than in non-pregnant women. Pregnant women who were in their first and second trimesters had significantly lower odds for singular Pb at or above the median blood level than non-pregnant women, whereas the odds were higher for pregnant women in their third trimester. Our study found higher odds of having at or above median levels of most metals and metal combinations among Mexican American women, non-Hispanic Black women, and women of other races compared to non-Hispanic White women.

By leveraging pharmacokinetic modeling within cross-sectional study designs, such as the NHANES, researchers can enhance their ability to quantify exposures accurately, estimate internal doses, understand individual variability, establish dose–response relationships, effectively adjust for confounders (such as age, sex, and smoking status), and explore toxicokinetics, ultimately improving the quality and interpretation of data obtained from these studies. Pharmacokinetics can significantly complement NHANES data by providing insights into how a chemical behave in the body across different populations (life stages modeling, pregnancy modeling, pediatric modeling, populations or individuals modeling). NHANES data includes information on environmental cumulative exposures and nutritional status (no exposure route, doses), which can affect the chemical ADME. PK models can incorporate these factors to provide a more comprehensive understanding of chemical behavior. More studies on metal combinations during pregnancy, particularly in each trimester, and in more understudied populations, are needed. Future work should focus on understanding the interactions within metal mixtures during pregnancy by employing prospective study designs and identifying possible environmental risk factors associated with adverse birth outcomes to improve maternal and infant health. Additionally, increasing awareness of exposures to multiple toxic metals and implementing targeted risk-reduction strategies in communities with elevated exposure levels may help lower the likelihood of exposure among pregnant women and their fetuses.

## Figures and Tables

**Table 1:** Specific unique combinations of cadmium (Cd), mercury (Hg), and lead (Pb) detected at or above the respective median concentrations in blood * among U.S. women aged 20–44, National Health and Nutrition Examination Survey (NHANES), 1999–2018.

Metal Combination	Blood Level			
	Non-Pregnant (N = 8855)		Pregnant (N = 1297)	
	N	Weighted Prevalence ^ % (95% CI)	N	Weighted Prevalence % (95% CI)
None	1268	17.4 (16.1–18.7)	256	22.9 (19.0–26.8)
Cd	847	10.0 (9.2–10.8)	152	14.7 (11.2–18.2)
Hg	1057	13.4 (12.3–14.5)	185	19.2 (15.0–23.3)
Pb	638	6.5 (5.8–7.2)	104	5.5 (4.1–6.9)
Cd/Hg	706	7.6 (6.8–8.4)	108	7.8 (6.0–9.6)
Cd/Pb	1465	15.6 (14.4–16.8)	197	9.8 (7.4–12.2)
Hg/Pb	856	9.4 (8.5–10.3)	108	9.2 (6.5–11.8)
Cd/Hg/Pb	2018	20.0 (18.8–21.3)	187	11.0 (8.7–13.2)

* Metal median blood concentrations: Cd = 0.3 ug/L, Hg = 0.76 ug/L, Pb = 0.73 ug/dL. ^ Weighted prevalence is for the non-institutionalized U.S. population; denominators are 8855 and 1297 for non-pregnant and pregnant, respectively.

**Table 2:** Specific unique combinations of cadmium (Cd), mercury (Hg), and lead (Pb) detected at or above the respective median concentrations in blood * among U.S. women aged 20–44, National Health and Nutrition Examination Survey (NHANES), 1999–2012.

Metal Combination	Non-Pregnant (N = 6966)		First and Second Trimesters (N = 615)		Third Trimester (N = 394)	
	N	Weighted Prevalence ^ % (95% CI)	N	Weighted Prevalence % (95% CI)	N	Weighted Prevalence % (95% CI)
None	985	15.3 (13.9–16.6)	150	26.4 (19.3–33.4)	83	21.7 (15.0–28.3)
Cd	779	11.7 (10.6–12.8)	102	14.9 (10.7–19.1)	61	16.5 (9.6–23.3)
Hg	817	12.7 (11.6–13.7)	89	19.9 (13.5–26.2)	52	20.1 (12.8–27.5)
Pb	466	5.9 (5.2–6.7)	38	3.4 (2.1–4.8)	39	9.9 (5.9–14.0)
Cd/Hg	643	8.7 (7.8–9.7)	57	9.7 (5.7–13.7)	35	6.9 (3.2–10.5)
Cd/Pb	1208	16.8 (15.3–18.2)	84	11.4 (7.1–15.7)	55	6.8 (4.2–9.4)
Hg/Pb	554	7.9 (7.0–8.9)	30	5.3 § (1.4–9.2)	25	6.2 (3.6–8.8)
Cd/Hg/Pb	1514	21.0 (19.5–22.5)	65	9.0 (5.1–12.9)	44	11.9 (6.0–17.9)

* Metal and median blood concentrations: Cd = 0.3 ug/L, Hg = 0.81 ug/L, Pb = 0.83 ug/dL. § Relative standard error ≥30%; ^ Weighted prevalence is for the non-institutionalized U.S. population; denominators are 6966, 615, and 394 for non-pregnant, first and second trimesters, and third trimester, respectively.

**Table 3:** Multinomial logistic regression for prevalent unique combinations * of blood cadmium (Cd), mercury (Hg), and lead (Pb) (“none” as reference) among U.S. women † aged 20–44, National Health and Nutrition Examination Survey (NHANES), 1999–2018.

Variable	Cd	Hg	Pb	Cd/Hg	Cd/Pb	Hg/Pb	Cd/Hg/Pb
Pregnant							
No	Reference	Reference	Reference	Reference	Reference	Reference	Reference
Yes	1.48 (1.02–2.15)	1.06 (0.74–1.51)	0.68 (0.48–0.97)	0.94 (0.63–1.39)	0.68 (0.47–0.98)	0.77 (0.51–1.17)	0.49 (0.36–0.69)
Age (yrs)							
20–29	Reference	Reference	Reference	Reference	Reference	Reference	Reference
30–44	1.30 (1.04–1.61)	0.98 (0.81–1.19)	1.44 (1.10–1.87)	1.57 (1.23–2.00)	2.51 (1.99–3.16)	1.90 (1.48–2.44)	3.04 (2.40–3.84)
Race/Ethnicity							
Non-Hispanic Whites	Reference	Reference	Reference	Reference	Reference	Reference	Reference
Mexican Americans	1.57 (1.19–2.08)	1.25 (0.91–1.71)	2.97 (2.08–4.24)	2.41 (1.62–3.58)	2.42 (1.75–3.33)	1.77 (1.27–2.47)	2.08 (1.48–2.93)
Non-Hispanic Blacks	1.22 (0.92–1.61)	1.74 (1.33–2.28)	1.34 (0.91–1.97)	2.31 (1.67–3.19)	1.15 (0.85–1.56)	1.63 (1.17–2.27)	2.82 (2.07–3.83)
Other races ^	1.52 (1.10–2.11)	2.10 (1.53–2.88)	1.34 (0.91–1.97)	4.09 (2.77–6.02)	2.20 (1.59–3.05)	1.77 (1.25–2.52)	5.05 (3.64–7.00)
Poverty income ratio							
≤1.85	Reference	Reference	Reference	Reference	Reference	Reference	Reference
>1.85–3.5	0.65 (0.48–0.86)	1.15 (0.90–1.49)	0.80 (0.60–1.08)	1.40 (1.03–1.89)	0.65 (0.50–0.83)	1.01 (0.77–1.33)	0.95 (0.76–1.20)
>3.5	0.88 (0.64–1.20)	2.07 (1.59–2.68)	1.32 (0.95–1.82)	2.52 (1.82–3.51)	0.84 (0.62–1.14)	2.47 (1.89–3.22)	2.24 (1.75–2.86)
Serum cotinine level (ng/mL)							
<0.015	Reference	Reference	Reference	Reference	Reference	Reference	Reference
0.015–<10	1.35 (1.01–1.80)	1.11 (0.88–1.39)	1.59 (1.15–2.20)	1.19 (0.86–1.63)	2.25 (1.68–3.01)	1.81 (1.32–2.47)	2.44 (1.86–3.19)
≥10	34.3 (21.1–55.9)	1.02 (0.54–1.91)	2.76 (1.33–5.69)	20.7 (12.7–33.8)	96.9 (59.4–158.1)	2.12 (1.18–3.83)	60.1 (37.3–96.7)

* Detected at or above the median concentration among U.S. women aged 20–44; † Sample size n = 9301 (8109 non-pregnant women, 1192 pregnant women). ^ Including races other than non-Hispanic Whites, Mexican Americans, non-Hispanic Blacks, but including multi-racial.

## Data Availability

The data presented in this study are openly available in the CDC NHANES site at https://wwwn.cdc.gov/nchs/nhanes/.

## Supplementary Materials

The following supporting information can be downloaded at: https://www.mdpi.com/article/10.3390/jox15020038/s1, Table S1: Demographic variables of the blood metal datasets for U.S. women aged 20–44 years, by pregnancy status, National Health and Nutrition Examination Survey (NHANES), 1999–2018; Table S2: Detection rates, geometric means, and medians of blood cadmium (Cd), mercury (Hg), and lead (Pb) for the U.S. women aged 20–44 years by pregnancy status, National Health and Nutrition Examination Survey (NHANES), 1999–2018; Table S3: Specific unique combinations of cadmium (Cd), mercury (Hg), and lead (Pb) detected at or above the respective median concentrations in the urine* among U.S. women aged 20-44, National Health and Nutrition Examination Survey (NHANES), 2003–2018; Table S4a: Level of Detection (LOD) for Cd, Hg, and Pb in blood by survey cycle; Table S4b: Level of Detection (LOD) for Cd, Hg, and Pb in urine by survey cycle; Figure S1: Distribution of urinary metal data by pregnancy status.
