# Supplementary material for: Prevalence of Exposure to Environmental Metal Mixtures Among Pregnant Women in the United States National Health and Nutrition Examination Survey (NHANES) 1999–2018
Source: J Xenobiot. 2025 Mar 1;15(2):38. doi: 10.3390/jox15020038 (PMC11932210; doi:10.3390/jox15020038)

List of Tables

Table		Page
Table S1	Demographic variables of the blood metal datasets for U.S. women aged 20-44 years, by pregnancy status, National Health and Nutrition Examination Survey (NHANES), 1999–2018	3
Table S2	Detection rates, geometric means, and medians of blood cadmium (Cd), mercury (Hg), and lead (Pb) for the U.S. women aged 20–44 years by pregnancy status, National Health and Nutrition Examination Survey (NHANES), 1999–2018	3
Table S3	Specific unique combinations of cadmium (Cd), mercury (Hg), and lead (Pb) detected at or above the respective median concentrations in the urine* among U.S. women aged 20-44, National Health and Nutrition Examination Survey (NHANES), 2003–2018	4
Table S4a	Level of Detection (LOD) for Cd, Hg, and Pb in blood by survey cycle	4
Table S4b	Level of Detection (LOD) for Cd, Hg, and Pb in urine by survey cycle	4

List of Figures

Figure		Page
Figure S1	Distribution of urinary metal data by pregnancy status	5

Table S1. Demographic variables of the blood metal datasets for U.S. women aged 20-44 years, by pregnancy status, National Health and Nutrition Examination Survey (NHANES), 1999–2018.

Variable	NHANES 1999–2018		
	Non-pregnant N (%)^	Pregnant N (%)	p-value*
N	8855	1297	
**Age (yrs)**			
Mean	32.4	28.4	
20–29	3331 (38.0)	825 (59.6)	<0.0001*
30–44	5524 (62.0)	472 (40.4)	
**Race**			
Mexican American	1800 (10.4)	353 (14.4)	
Non-Hispanic Black	1906 (13.2)	200 (15.8)	0.0005*
Non-Hispanic White	3500 (61.3)	562 (53.0)	
Other Race	1649 (15.1)	182 (16.8)	
**Poverty income ratio (PIR)**			
0 ≤ PIR ≤1.85	4064 (39.7)	573 (39.4)	
1.85 < PIR ≤3.50	1934 (25.7)	267 (22.6)	0.3057
3.50 < PIR	2210 (34.6)	370 (38.0)	
**Serum cotinine levels (ng/mL)**			
<0.015	1862 (23.4)	401 (30.9)	
0.015–9.999	4760 (51.4)	724 (55.6)	<0.0001*
≥10	2128 (25.2)	154 (13.5)	

*Chi-square test: p<0.05 is statistically significant.

^N is unweighted; % is weighted

Table S2. Detection rates, geometric means, and medians of blood cadmium (Cd), mercury (Hg), and lead (Pb) for the U.S. women aged 20–44 years by pregnancy status, National Health and Nutrition Examination Survey (NHANES), 1999–2018.

Chemical analytes	Non-pregnant				Pregnant			
	N	Detection rate (%)	Geometric mean	Median	N	Detection rate (%)	Geometric mean	Median
Cd	8863	81.1	0.34 (0.33–0.36)	0.30 (0.30–0.30)	1298	70.4	0.27 (0.25–0.29)	0.25 (0.21–0.28)
Hg	8855	89.1	0.81 (0.78–0.84)	0.77 (0.72–0.80)	1297	87.6	0.70 (0.63–0.77)	0.72 (0.60–0.80)
Pb	8863	99.3	0.76 (0.75–0.78)	0.75 (0.72–0.77)	1298	96.0	0.61 (0.57–0.66)	0.60 (0.56–0.62)

Geometric means & medians for Cd & Hg are in ug/L; Pb is in ug/dL

Table S3. Specific unique combinations of cadmium (Cd), mercury (Hg), and lead (Pb) detected at or above the respective median concentrations in the urine* among U.S. women aged 20-44, National Health and Nutrition Examination Survey (NHANES), 2003–2018.

**Metal combination**	**Urine level**			
	**Non-pregnant (N = 2756)**		**Pregnant (N = 285)**	
	**N**	**Weighted prevalence % (95% CI)**	**N**	**Weighted prevalence % (95% CI)**
None	438	20.8 (18.6–23.0)	22	12.7 (6.7–18.6)
Cd	255	9.9 (8.5–11.3)	14	8.5 (5.6–11.5)
Hg	225	9.4 (7.9–10.8)	18	12.7 (6.8–18.6)
Pb	243	8.7 (7.7–9.7)	51	15.1 (8.3–21.9)
Cd/Hg	303	10.7 (9.4–12.0)	17	4.5 (1.9–7.0)
Cd/Pb	357	10.7 (9.5–12.0)	30	8.3 (5.5–11.1)
Hg/Pb	287	10.9 (9.4–12.4)	67	18.0 (12.5–23.5)
Cd/Hg/Pb	648	18.9 (16.9–20.8)	66	20.2 (14.6–25.9)

*Metal and median urine concentrations: Cd = 0.167 ug/L, Hg = 0.333 ug/L, and Pb = 0.304 ug/L.

Table S4a. Level of Detection (LOD) for Cd, Hg, and Pb in blood by survey cycle.

**Metal**	**NHANES 1999-2000**	**NHANES 2001-2002**	**NHANES 2003-2004**	**NHANES 2005-2006**	**NHANES 2007-2008**	**NHANES 2009-2010**	**NHANES 2011-2012**	**NHANES 2013-2014**	**NHANES 2015-2016**	**NHANES 2017-2018**
**Cd**	0.3	0.3	0.14	0.2	0.2	0.2	0.16	0.1	0.1	0.1
**Hg**	NA	NA	0.2	0.33	0.33	0.33	0.16	0.28	0.28	0.28
**Pb**	0.3	0.3	0.28	0.25	0.25	0.25	0.25	0.07	0.07	0.07

Table S4b. Level of Detection (LOD) for Cd, Hg, and Pb in urine by survey cycle.

**Metal**	**NHANES 2003-2004**	**NHANES 2005-2006**	**NHANES 2007-2008**	**NHANES 2009-2010**	**NHANES 2011-2012**	**NHANES 2013-2014**	**NHANES 2015-2016**	**NHANES 2017-2018**
**Cd**	0.06	0.042	0.042	0.042	0.056	0.036	0.036	0.036
**Hg**	0.14	0.11	0.08	0.08	0.05	0.13	0.13	0.13
**Pb**	0.33	0.1	0.1	0.1	0.08	0.03	0.03	0.03

Figure S1. Distribution of urinary metal data by pregnancy status. A. Cadmium, B. Lead, C. Mercury

A

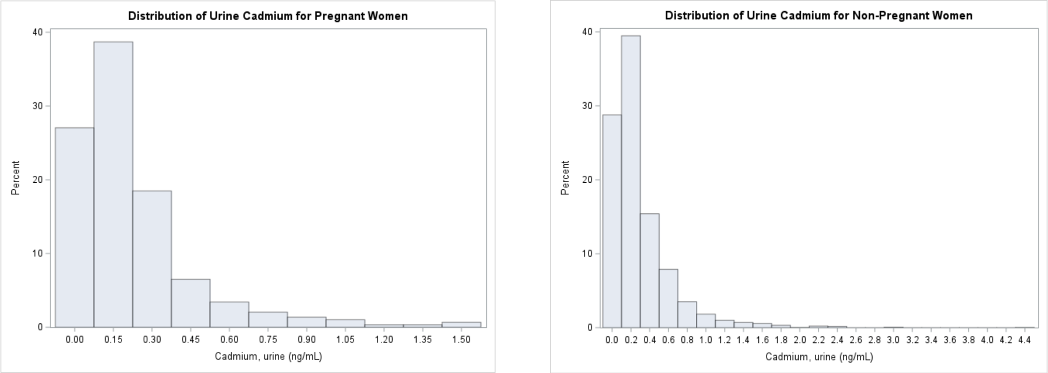

B

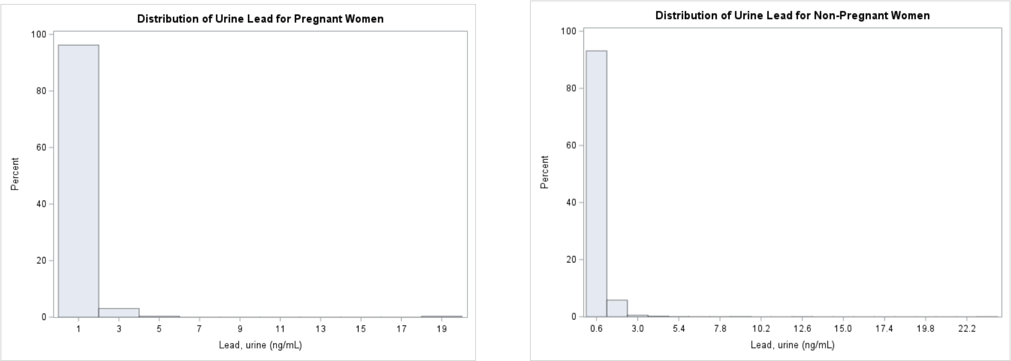

C

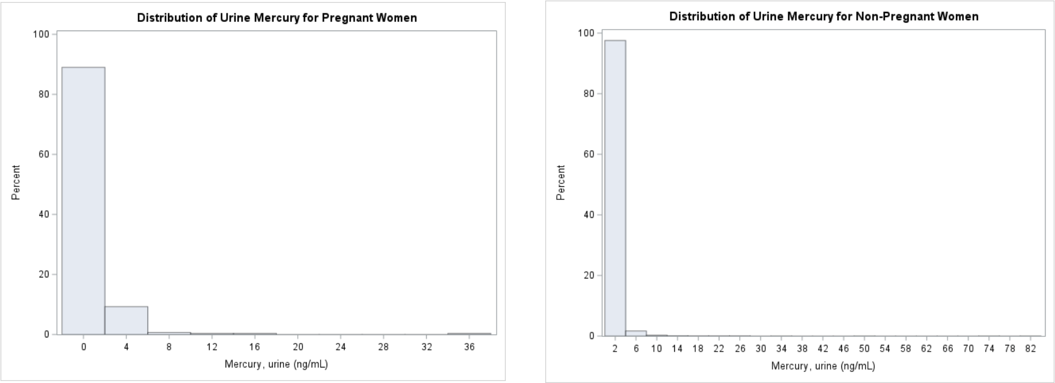

Supplement: Supplementary file 1 [file jox-15-00038-s001.zip › jox-3394903-supplementary.pdf]
